# Association of homocysteine with carotid-femoral pulse wave velocity in a southern Chinese population

**DOI:** 10.18632/aging.102416

**Published:** 2019-11-11

**Authors:** Tingjun Wang, Guoyan Xu, Xiaoqi Cai, Jin Gong, Qunfang Xie, Liangdi Xie

**Affiliations:** 1 Department of General Medicine, The First Affiliated Hospital of Fujian Medical University, Fuzhou, Fujian 350005, People’s Republic of China; 2 Fujian Hypertension Research Institute, Fuzhou, Fujian 350005, People’s Republic of China; 3 Department of Geriatric Medicine, The First Affiliated Hospital of Fujian Medical University, Fuzhou, Fujian 350005, People’s Republic of China

**Keywords:** arterial stiffness, carotid-femoral pulse wave velocity, elderly, homocysteine, hypertension

## Abstract

This study aimed to investigate whether plasma homocysteine levels were associated with carotid-femoral pulse wave velocity (cfPWV), a golden standard of arterial stiffness, in a population from southern China. A cross-sectional study was conducted on 713 patients admitted to the First Affiliated Hospital of Fujian Medical University from February 2016 to August 2017. They were divided into four groups based on gender-specific quartile of homocysteine levels. Age, cfPWV, uric acid levels, and percentage of hypertension increased with ascending quartiles. The duration of hypertension and systolic blood pressure were higher in the highest quartile than in the lowest quartile. Pearson’s correlation analysis and multivariate regression showed a correlation of homocysteine levels with cfPWV. A nearly twofold increased risk of cfPWV ≥10 m/s was observed in the highest quartile compared with the lowest quartile (in the highest quartile: odds ratio = 2.917, 95% confidence interval: 1.635–5.202, *P* < 0.001). After stratification, this correlation was present in both sexes, in patients aged over 65 years, and those with hypertension. The plasma homocysteine levels were independently associated with cfPWV in the population from southern China, especially in the elderly and those with hypertension.

## INTRODUCTION

Arterial stiffness is characterized by loss of elastin fibers and accumulation of stiffer collagen fibers in the media of large arteries, reflecting the result of arterial wall remodeling [1]. Generally, arterial stiffness progresses with aging and now considered as a hallmark of vascular aging [2, 3]. Patients with arterial stiffness are at high risk of cardiovascular disease and declined cognition [4, 5]. Arterial stiffness may be assessed by different methods, of which the measurement of carotid-femoral pulse wave velocity (cfPWV) is proved to be simple, validated, and reproducible. cfPWV is well recognized as a golden standard for assessing arterial stiffness [6]. Increased pulse wave velocity (PWV) has been shown to be associated with traditional cardiovascular risk factors, such as aging, hypertension, and diabetes. Furthermore, arterial stiffness progresses more rapidly with increasing numbers of traditional risk factors [7–9].

Currently, some risk factors not traditionally recognized have also been linked to arterial stiffness [10, 11]. Homocysteine, a sulfur-containing amino acid and an intermediate product of methionine metabolism, is a nontraditional risk factor for cardiovascular disease. Deficiency of metabolic enzymes and co-factors of homocysteine, such as folic acid and vitamin B, may lead to increased homocysteine levels in humans and mice [12, 13]. In the last decades, the relationship between homocysteine levels and arterial stiffness has been repeatedly investigated; however, the results are controversial [14, 15]. This inconsistency may be due to the differences in studied populations and methods used for assessing arterial stiffness. A positive association of homocysteine with PWV has been reported in a population from Beijing in the northern part of China [16]. However, the existence of this relationship in the general population of China has not been verified.

This study aimed to investigate the relationship between plasma homocysteine levels and cfPWV in a population from southern China.

## RESULTS

The clinical characteristics of all 713 patients based on the gender-specific quartile of homocysteine levels are shown in Table 1. The mean age of patients was 59 ± 12 years, 438 (61.4%) were male, and 275 (38.6%) were female. The plasma homocysteine level ranged from 0.5 to 50 μmol/L, and the median was 11.2 μmol/L in male patients and 8.70 μmol/L in female patients. From the lowest to the highest quartile of homocysteine levels, the median and range were as follows: for male patients, the lowest quartile was 8.33 (0.72–9.35) μmol/L, second quartile 10.23 (9.36–11.11) μmol/L, third quartile 12.08 (11.12–13.72) μmol/L, and highest quartile 15.99 (13.73–50.00) μmol/L; for female patients, the lowest quartile was 6.19 (0.50–7.34) μmol/L, second quartile 8.00 (7.35–8.68) μmol/L, third quartile 9.61 (8.70–11.12) μmol/L, and highest quartile 13.00 (11.13–34.71) μmol/L. Of all the patients, 489 (68.6%) were diagnosed with hypertension and 166 (23.3%) with diabetes.

**Table 1:** Comparison of clinical characteristics of patients in the quartile of homocysteine levels (*n* = 713).

**Variables**	**Quartile 1**	**Quartile 2**	**Quartile 3**	**Quartile 4**	***F*/*χ^2^***	***P***
Homocysteine (μmol/L) Male	8.33 (7.58–8.90)	10.23 (9.85–10.60)^*^	12.08 (11.59–12.77)^*^	15.99 (14.72–19.45)^*^	318.807	<0.001
Female	6.19 (5.33–6.76)	8.00 (7.65–8.20)^*^	9.61 (9.22–10.18)^*^	13.00 (11.74–15.11)^*^	200.015	<0.001
Age (year)	56.2 ± 10.3	56.7 ± 11.2	59.9 ± 10.7^*^	63.2 ± 14.2^*^	13.918	<0.001
Current smoking [*n* (%)]	38 (21.5)	55 (30.9)	56 (31.3)	64 (35.8)	9.182	0.027
Body mass index (kg/m^2^)	24.6 ± 2.8	24.3 ± 4.4	25.0 ± 3.1	25.5 ± 3.8	3.383	0.018
Hypertension [*n* (%)]	99 (55.9)	122 (68.3)^*^	122 (68.9)^*^	146 (81.6)^*^	27.162	<0.001
Duration of hypertension (year)	1.00 (0–10.00)	2.50 (0–10.00)	3.00 (0–8.00)	6.00 (5.00–13.00)^*^	9.765	<0.001
Systolic blood pressure (mm Hg)	129.9 ± 16.8	132.9 ± 18.3	131.6 ± 19.1	138.0 ± 21.4^*^	5.963	0.001
Diastolic blood pressure (mm Hg)	80.7 ± 11.4	80.2 ± 12.1	79.2 ± 12.5	79.0 ± 13.0	0.792	0.499
Heart rate (bp)	73.0 ± 11.9	71.2 ± 11.0	72.1 ± 11.0	72.3 ± 12.2	0.722	0.539
Diabetes [*n* (%)]	37 (20.9)	41 (23.0)	41 (22.9)	47 (26.3)	1.468	0.690
Fasting plasma glucose (mmol/L)	5.83 ± 1.70	5.79 ± 1.56	5.64 ± 1.65	5.55 ± 1.27	1.214	0.304
Total cholesterol (mmol/L)	4.66 ± 0.94	4.65 ± 1.05	4.75 ± 1.13	4.75 ± 1.13	0.414	0.743
Triglyceride (mmol/L)	1.25 (0.87–1.77)	1.29 (0.91–1.85)	1.29 (0.93–1.74)	1.43 (1.06–1.83)	2.546	0.055
HDL-C (mmol/L)	1.27 ± 0.33	1.25 ± 0.33	1.28 ± 0.40	1.21 ± 0.34	1.247	0.292
LDL-C (mmol/L)	2.89 ± 0.92	2.82 ± 0.91	2.99 ± 1.06	2.98 ± 1.01	1.174	0.319
eGFR (mL/min∙1.73 m^2^)	99.0 ± 40.6	103.3 ± 35.5	98.4 ± 36.3	107.4 ± 47.9	1.169	0.321
Uric acid (μmol/L)	346.8 ± 91.7	360.9 ± 89.7	379.2 ± 92.2^*^	413.9 ± 113.7^*^	15.214	<0.001
cfPWV (m/s)	8.73 ± 1.61	8.88 ± 1.49	9.33 ± 1.69^*^	10.4 ± 4.82^*^	12.363	<0.001
Use of ACEI/ARB [*n* (%)]	107 (60.5)	111 (62.4)	102 (57.0)	128 (71.5)	1.313	0.236
Use of aspirin [*n* (%)]	92 (52.0)	91 (51.1)	88 (49.2)	102 (57.6)	1.946	0.123
Use of statin [*n* (%)]	122 (68.9)	109 (61.2)	98 (54.7)	126 (70.4)	1.264	0.285

Data are expressed as mean ± standard or median (25^th^–75^th^). ACEI: Angiotensin-converting enzyme inhibitor; ARB: angiotensin receptor blocker; cfPWV, carotid-femoral pulse wave velocity; eGFR, estimated glomerular filtration rate; HDL-C, high-density lipoprotein cholesterol; LDL-C, low-density lipoprotein cholesterol. ^*^P < 0.016 vs. quartile 1.

The patients in a higher quartile of homocysteine levels were older. Hypertension was frequently observed and uric acid levels increased in the higher quartile. The duration of hypertension [6 (5–13) vs 1 (0–10) years], systolic blood pressure [(138.0 ± 21.4) vs (129.9 ± 16.8) mm Hg] were higher in the highest quartile than in the lowest quartile (*P* < 0.016). With ascending quartiles of homocysteine levels, a trend of increasing cfPWV was observed (8.73 ± 1.61, 8.88 ± 1.49, 9.33 ± 1.69, and 10.40 ± 4.82 m/s, *P* < 0.001). No significant between-group differences were found in diastolic blood pressure, fasting plasma glucose, total cholesterol, triglyceride, high-density lipoprotein cholesterol (HDL-C), low-density lipoprotein cholesterol (LDL-C), estimated glomerular filtration rate (eGFR), percentage of diabetes, and use of medication.

**Figure 1:**
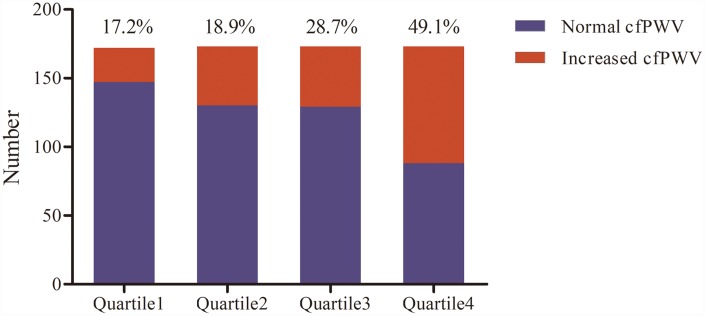
**Percentage of increased carotid-femoral pulse wave velocity (cfPWV) in the quartile of homocysteine levels (n = 713).**

Percentages of cfPWV ≥10 m/s in different quartiles of homocysteine levels are demonstrated in Figure 1. cfPWV ≥10 m/s was more frequently present in the higher quartile. A striking increase in the percentage of cfPWV ≥10 m/s was found from the third quartile to the highest quartile (17.2%, 18.9%, 28.7%, and 49.1%, *χ*_trend_ = 54.097, *P* < 0.001).

**Figure 2:**
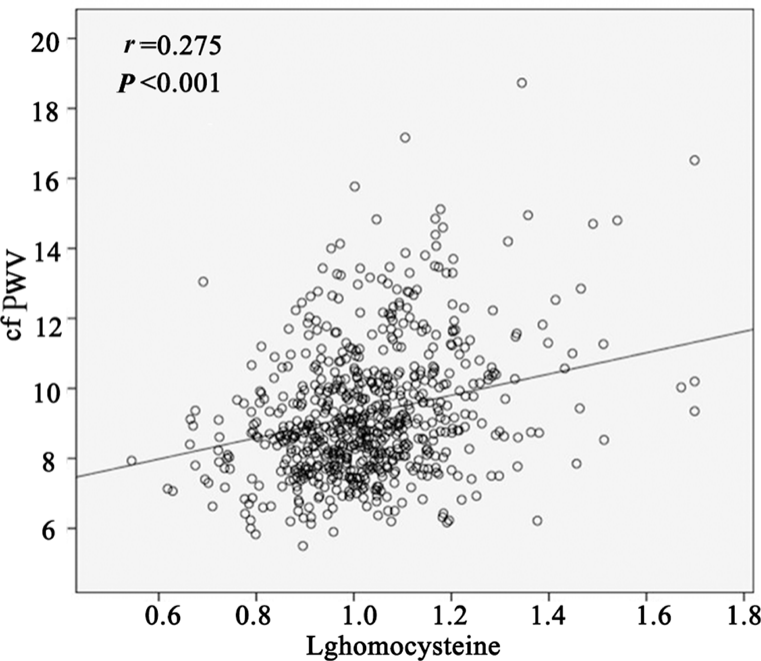
**Correlation of Lg homocysteine with carotid-femoral pulse wave velocity (cfPWV) in scatter diagram (n = 713).**

When analyzed as a continuous variable, the log-transformed homocysteine level correlated with cfPWV (*r* = 0.275, *P* < 0.001) (Table 2 and Figure 2), even after adjusting for age, systolic blood pressure, heart rate, Lg triglyceride, and uric acid level (*β* = 0.147, adjusted *r* = 0.175, *P* = 0.001). Standardized regression equation: cfPWV = –3.638 + 0.429 × Age + 0.283 × Systolic blood pressure + 0.169 × Heart rate + 0.147 × Lg homocysteine + 0.124 × Lg triglyceride + 0.041 × Uric acid; adjusted *R^2^* = 0.459 (Table 3).

**Table 2:** Pearson’s correlation analysis for carotid-femoral pulse wave velocity (*n* = 713).

**Variables**	***r***	***P***
Sex (male = 0, female = 1)	0.016	0.678
Age	0.322	<0.001
Current smoking (N = 0, Y = 1)	–0.010	0.798
Body mass index	0.144	<0.001
Duration of hypertension	0.271	<0.001
Systolic blood pressure	0.274	<0.001
Diastolic blood pressure	0.025	0.506
Heart rate	0.038	0.332
Fasting plasma glucose	0.064	0.101
Total cholesterol	–0.087	0.025
Lg triglyceride	0.028	0.469
HDL-C	–0.084	0.031
LDL-C	–0.062	0.106
eGFR	0.137	0.005
Uric acid	0.068	0.078
Lg homocysteine	0.275	<0.001

eGFR: Estimated glomerular filtration rate; HDL-C: high-density lipoprotein cholesterol; LDL-C: low-density lipoprotein cholesterol.

**Table 3:** Stepwise multivariate linear regression analysis for carotid-femoral pulse wave velocity.

**Variables**	***B***	***E***	***β***	***r***	***P***
Age	0.061	0.007	0.429	0.487	<0.001
Systolic blood pressure	0.029	0.006	0.283	0.351	<0.001
Heart rate	0.024	0.007	0.169	0.186	<0.001
Lg homocysteine	1.812	0.560	0.147	0.175	0.001
Lg triglyceride	0.999	0.485	0.124	0.141	0.004
Uric acid	0.001	0.002	0.041	0.044	0.024

*R^2^* = 0.465, adjusted *R^2^* = 0.459. Candidate variables: age, gender, current smoking, body mass index, duration of hypertension, systolic blood pressure, diastolic blood pressure, heart rate, diabetes, fasting plasma glucose, total cholesterol, Lg triglyceride, high-density lipoprotein cholesterol, low-density lipoprotein cholesterol, estimated glomerular filtration rate, Lg homocysteine, uric acid, and use of medication.

Increasing odds ratios (ORs) for cfPWV ≥10 m/s were displayed from the lowest quartile to the highest quartile of homocysteine levels. The graded association remained statistically significant after adjusting for demographic data, anthropometric data, laboratory indicators, and use of medication. A nearly twofold increased risk of cfPWV ≥10 m/s was observed in the highest quartile compared with the lowest quartile (highest quartile: OR = 2.917, 95% CI: 1.635–5.202, *P* < 0.001) (Table 4).

**Table 4:** Odd ratios of the quartile of homocysteine levels for increased carotid-femoral pulse wave velocity in univariate and multivariate logistic regression analyses.

**Homocysteine**	**Univariate**			**Multivariate**		
	**OR**	**95% CI**	***P***	**OR**	**95% CI**	***P***
Quartile 1	1	Reference		1	Reference	
Quartile 2	1.126	0.643–1.972	0.678	1.057	0.656–1.611	0.532
Quartile 3	1.944	1.152–3.280	0.013	1.797	0.988–3.235	0.051
Quartile 4	4.659	2.816–7.708	<0.001	2.917	1.635–5.202	<0.001

Univariate: no adjustment. Multivariate: adjustment for age, gender, current smoking, body mass index, duration of hypertension, systolic blood pressure, diastolic blood pressure, heart rate, diabetes, fasting plasma glucose, total cholesterol, triglyceride, high-density lipoprotein cholesterol, low-density lipoprotein cholesterol, estimated glomerular filtration rate, uric acid level, and use of medication.

**Figure 3:**
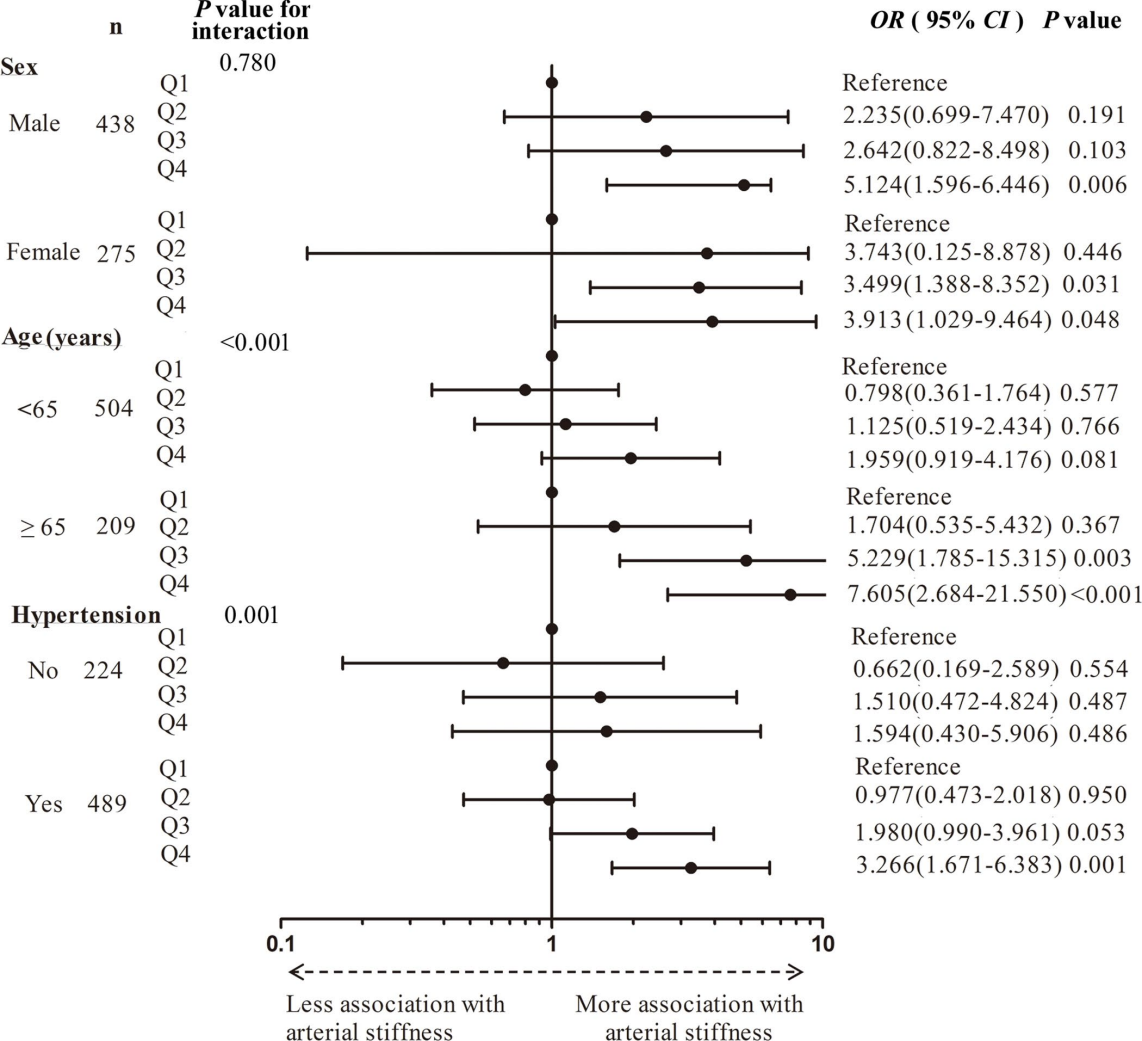
**Forest plot of the quartile of homocysteine levels for increased carotid-femoral pulse wave velocity in stratified analysis by gender, age, and presence of hypertension.** Q, Quartile.

Before further stratification, interaction analyses were performed. Age and presence of hypertension were found to significantly modify the association of the quartile of homocysteine levels with cfPWV ≥10 m/s. After gender stratification, a significant association between the quartile of homocysteine levels and cfPWV ≥10 m/s was observed in both male and female patients. This association appeared to be more prominent in male patients. After age stratification, a significant association was present only in patients older than 65 years, but not those younger than 65 years. The elderly in the highest quartile of homocysteine levels had a 6.6-fold increased risk of cfPWV ≥10 m/s compared with those in the lowest quartile (highest quartile: OR *=* 7.605, 95% CI: 2.684–21.550, *P* < 0.001). After stratification of hypertension, a significant association persisted in patients with hypertension. Patients in the highest quartile had an almost 2.3-fold increased risk of cfPWV ≥10 m/s compared with those in the lowest quartile (highest quartile: OR = 3.266, 95% CI: 1.671–6.383, *P* = 0.001). However, the association became weak in patients without hypertension (Figure 3).

## DISCUSSION

In this study, the homocysteine levels were shown to be independently associated with cfPWV, the golden standard of arterial stiffness. Furthermore, this association was pronounced mainly in the elderly and patients with hypertension.

cfPWV has been recognized as an independent predictor of cardiovascular morbidity and mortality [17]. Increasing evidence indicates that cfPWV may be used as a parameter for better individualized treatment [18, 19]. Age, blood pressure, and blood pressure–related parameters are associated with PWV, as confirmed in a previous study [20, 21]. In addition, some nontraditional cardiovascular risk factors, such as uric acid levels, albuminuria, and homocysteine levels, have also been reported to contribute to increased PWV [11, 22].

In the last decades, homocysteine was found to be associated with PWV in western populations [14]. Recently, the association was also shown in a population from Beijing in the northern part of China [23]. However, PWV and homocysteine levels were known to vary with ethnicity and lifestyle [24–26]. Dietary profile and habit in southern Chinese are different from those in northern Chinese. Generally, the dietary folic acid level is higher in southern Chinese than in northern Chinese [27] Therefore, it may be speculated that homocysteine levels are lower in southern Chinese, as verified in the present study. The median of plasma homocysteine levels was 11.2 μmol/L in male patients and 8.70 μmol/L in female patients, which was lower than that in the population from Beijing [16]. However, for these lower levels of plasma homocysteine, the relationship between homocysteine levels and PWV remained unchanged.

Increased homocysteine level, termed hyperhomocysteinemia, is defined as plasma homocysteine levels greater than 15 μmol/L [28]. As a matter of fact, when plasma homocysteine levels are higher than 10 μmol/L, a dose–response relationship exists between homocysteine levels and cardiovascular disease [29]. Also, a 1.6-fold increase in the risk of cardiovascular disease has been reported for every 5 μmol/L increment of the plasma homocysteine level [30, 31]. The present study identified a positive association between homocysteine levels and cfPWV, and a marked increase in the percentage of cfPWV ≥10 m/s was observed at the homocysteine level of around 12 μmo/L in male patients and 9.5 μmo/L in female patients. This level was below the threshold of hyperhomocysteinemia. In this study, patients in the higher quartile of homocysteine levels were found to have more concomitant risk factors for cardiovascular disease. However, after adjusting for these traditional cardiovascular risk factors, the association remained unchanged. Hence, it was suggested that the association of plasma homocysteine levels with arterial stiffness was independent.

Previous studies examined the relationship between plasma homocysteine levels and cardiovascular disease. However, the conclusions remain controversial. Bortolotto et al. reported that cfPWV was higher in patients with higher homocysteine levels [32]. In contrast, Nakhai-Pour et al. found no independent association of homocysteine levels with PWV [14]. Even in two samples from the Framingham Heart Study, Levy et al. and Lieb et al. drew different conclusions about the relationship between these two parameters [33, 34]. This inconsistency might be due to the different characteristics of studied patients. The present study showed a positive association of plasma homocysteine levels with cfPWV only in patients older than 65 years and those with hypertension, which was consistent with some previous studies reporting a positive association only among patients with greater risks of cardiovascular disease, such as hypertension, diabetes, or chronic kidney disease, or elderly patients [35–38]. However, the mechanism underlying the interaction between homocysteine levels and other cardiovascular risk factors has not been fully clarified. Nevertheless, all these cardiovascular risk factors, including age, may lead to endothelial dysfunction, and the damage may make the arterial wall more susceptible to the deleterious effect of homocysteine [39, 40]. This may explain a positive association of plasma homocysteine levels with cfPWV only in patients older than 65 years and those with hypertension.

Although the positive association of plasma homocysteine levels with arterial stiffness was revealed, available evidence regarding the benefit of homocysteine-lowering therapies, such as supplementation of folic acid or vitamin B12, is inconsistent. The B-Vitamins for the Prevention of Osteoporotic Fractures study demonstrated that a 2-year supplementation of vitamin B12 and folic acid in elderly patients with hyperhomocysteinemia had no significant benefit on arterial stiffness [41]. In contrast, a Chinese study found that the combined use of folic acid and antihypertensive agents, compared with antihypertensive agents alone, might significantly reduce the risk of first stroke in patients with hypertension [42]. The benefit of homocysteine-lowering therapies against cardiovascular disease needs to be confirmed. The present study did not involve the use of homocysteine-lowering therapy; however, arterial stiffening was known to have very limited reversibility. Therefore, it is not surprising that short-term homocysteine-lowering therapies yielded null results in the elderly. The greatest benefit of homocysteine-lowering therapies can be achieved in patients with no sign of arterial stiffness, but with multiple concomitant cardiovascular risk factors.

A wide age range and a higher proportion of hypertension were unique aspects of this study, facilitating the stratified analysis. However, several potential concerns and limitations are worth mentioning. First, the sample size was relatively small. Second, no causal relationship could be determined because of the cross-sectional design of the study. Third, the population comprised inpatients, not community-based residents, thus decreasing the generalizability of the results. Finally, all patients were from Fujian province, and hence the conclusions drawn from this study could not be generalized to other populations. A prospective, long-term interventional study is needed to investigate the preventive effect of homocysteine-lowering therapy on the progression of arterial stiffness.

In conclusion, an independent relationship existed between homocysteine levels and cfPWV. The association was more pronounced in patients older than 65 years and those with hypertension. This study provided evidence for the association of plasma homocysteine levels with arterial stiffness in a population from southern China.

## MATERIALS AND METHODS

### Patients

This was a single-center, cross-sectional, observational study.

### Ethics statement

The procedures were in accordance with the Helsinki Declaration, and the protocol was approved by the Ethical Committee of the First Affiliated Hospital of Fujian Medical University. Informed consent was obtained from all patients.

A specific protocol was established before data collection, and trained physicians were responsible for the procedure. Patients were recruited from the inpatients consecutively admitted to the general medicine department of the First Affiliated Hospital of Fujian Medical University from February 1, 2016, to August 31, 2017. The reason for admission was to evaluate the patients as follows: routine health examination, or screening of hypertension-related or diabetes-related target organ damage, or treatment of coronary heart disease, peptic ulcer, Alzheimer disease, renal cyst, and so on. A total of 837 patients were included, of whom, 124 patients were excluded due to myocardial diseases, valvular heart disease, atrial fibrillation, serious arrhythmias, a history of acute myocardial infarction, stroke within the last 6 months, peripheral artery diseases, carotid artery occlusion or carotid sinus syndrome, serum creatinine (Scr) >2.5 mg/dL, chronic liver failure, autoimmune diseases, active malignancy, acute infection diseases, connective tissue diseases, use of folic acid and vitamin B, or pregnancy. Finally, 713 patients were enrolled in this study.

### Clinical data

As described in a previous study, all patients were interviewed on admission to the hospital, and information was collected regarding age, gender, cigarette smoking and drinking habits, family history, medical history of hypertension and diabetes, use of medications, and so on [43]. Current smoking was defined as consuming at least one cigarette per day for at least 6 months. According to the European Society of Cardiology (ESC)/European Society of Hypertension (ESH) guidelines for managing arterial hypertension, hypertension was defined as systolic blood pressure ≥140 mm Hg and/or diastolic blood pressure ≥90 mm Hg and/or taking antihypertensive medications [44]. Diabetes was defined as taking antihyperglycemic medications or establishing a new diagnosis of diabetes. The diagnosis of diabetes was based on the criteria recommended by the Chinese Diabetes Society in 2017 [45].

Height and body weight were recorded for patients in light clothes and without shoes. Height was recorded to the nearest 0.5 cm and body weight to 0.1 kg. Body mass index was calculated using the following formula: body mass index = body weight (kg)/height^2^ (m^2^). After patients rested for at least 5 min in the sitting position, the heart rate was taken and the blood pressure was measured using an automated sphygmomanometer (HBP-1300, Omron, Kyoto, Japan). The mean of three measurements was used for the subsequent analysis.

The venous blood sample was obtained after overnight fasting. Plasma homocysteine levels were estimated by chemiluminescence microparticle immunoassay according to the manufacturer’s protocol (Abbott GmbH & Co.KG, Wiesbaden-Delkenheim, Germany). The intra-variable coefficient was 3%, and the inter-variable coefficient was 5%. The patients were divided into four groups based on the gender-specific quartile of homocysteine levels. The levels of fasting plasma glucose, serum creatinine (Scr), total cholesterol, HDL-C, LDL-C, and uric acid were determined using an autoanalyzer (ADVIA 2400, Siemens, Germany). The eGFR was calculated according to the modification of diet in the renal disease equation: eGFR [mL/(min·1.73 m^2^)] =186 × [Scr (mmol/L)/88.41]^-1.154^ × (Age)^-0.203^ (× 0.742 female).

### Measurement of cfPWV

cfPWV was measured using Complior Analysis (Alam Medical, Saint-Quentin-Fallavier, France), as recommended by the Artery Society and the European Society of Hypertension Working Group on Vascular Structure and Function, as described previously by our team [6, 21]. Briefly, smoking, caffeine, and alcohol intake were prohibited 12 h before the measurement. The patients were asked to lie down in a supine position in a quiet room with a stable room temperature. Measurements were performed by a well-trained and experienced technician. The direct straight distance between the right common carotid artery and the right common femoral artery was measured using a tape, and the tape-measured distance multiplied by 0.8 was used as the pulse wave traveled distance. Then, two TY-360 pressure-sensitive transducers (Fukuda Denshi Co, Tokyo, Japan) were positioned at these two measurement sites, and two different pulse waves were obtained. This performance was repeated for at least 10 cardiac cycles. cfPWV was calculated according to the following formula: cfPWV (m/s) = carotid-femoral pulse wave travel distance (m)/travel time (s). The mean value of the two measurements was taken. If the difference between the two measurements was greater than 0.5 m/s, a third measurement was performed, and the median value of the three measurements was taken. Further, ≥10 m/s was used as the cutoff value of increased cfPWV.

### Statistical analysis

Continuous variables were expressed as mean ± standard deviation or median, and categorical variables were presented as absolute numbers or percentages. Homocysteine and triglyceride levels were logarithmically (log) transformed for further analysis because of skewed distribution. Variables were compared by one-way analysis of variance or chi-square test. The correlation between variables was assessed by Pearson’s correlation analysis and stepwise multivariate linear regression. Multivariate logistic regression was performed to identify the association of the quartile of homocysteine levels with cfPWV ≥10 m/s, and the corresponding OR was calculated for increased cfPWV. Statistical significance was accepted at *P* < 0.05 for individual data. For multiple comparisons, the threshold levels of significance were adjusted by simple Bonferroni’s correction. All the statistical analyses were performed using SPSS statistical software, version 19.0 (IL, USA).
